# Improving clinical trial transparency at UK universities: Evaluating 3 years of policies and reporting performance on the European Clinical Trial Register

**DOI:** 10.1177/17407745211071015

**Published:** 2022-02-16

**Authors:** Sarai Mirjam Keestra, Florence Rodgers, Sophie Gepp, Peter Grabitz, Till Bruckner

**Affiliations:** 1 Department of Epidemiology and Data Science, Amsterdam UMC, University of Amsterdam, Amsterdam, The Netherlands; 2 School of Medicine, Imperial College London, London, UK; 3 Charité– Universitätsmedizin Berlin, Berlin, Germany; 4 BIH QUEST Center, Berlin, Germany; 5 TranspariMED, Bristol, UK

**Keywords:** Clinical trial transparency, publication bias, universities, research waste, missing trial results, research governance

## Abstract

**Background:**

January 2019, the House of Commons’ Science and Technology Committee sent letters to UK universities admonishing them to achieve compliance with results reporting requirements for Clinical Trials of Investigative Medicinal Products by summer 2019. This study documents changes in the clinical trial policies and Clinical Trials of Investigative Medicinal Product reporting performance of 20 major UK universities following that intervention.

**Methods:**

Freedom of Information requests were filed in June 2018 and June 2020 to obtain clinical trial registration and reporting policies covering both Clinical Trials of Investigative Medicinal Products and all other clinical trials. Two independent reviewers assessed policies against transparency benchmarks based on World Health Organization best practices. To evaluate universities’ trial reporting performance, we used a public online tracking tool, the European Union Trials Tracker, which assesses universities’ compliance with regulatory Clinical Trials of Investigative Medicinal Product disclosure requirements on the European Clinical Trial Register. Specifically, we evaluated whether universities were adhering to the European Union requirement to post summary results on the trial registry within 12 months of completion.

**Results:**

Mean policy strength increased from 2.8 to 4.9 points (out of a maximum of 7 points) between June 2018 and June 2020. In October 2018 the average percentage of due Clinical Trials of Investigative Medicinal Products that had results available on the European trial registry across university sponsors included in the cohort was 29%. By June 2021, this had increased to 91%, with 5 universities achieving a reporting performance of 100%. All 20 universities reported more than 70% of their due trial results on the European trial registry.

**Interpretation:**

Political pressure appears to have a significant positive impact on UK universities’ clinical trial reporting policies and performance. Similar approaches could be used to improve reporting performance for other types of sponsors, other types of trials, and in other countries.

## Background

Clinical trial transparency is at the foundation of evidence-based medicine and an ethical obligation. Article 36 of the Helsinki Declaration on Ethical Research Involving Human Subjects states that ‘researchers have a duty to make publicly available the results of their research on human subjects and are accountable for the completeness and accuracy of their reports’.^1^ Furthermore, according to the World Health Organization (WHO)^2^ best practices, the results of all interventional clinical trials should be uploaded onto trial registries within 12 months of trial completion; the UK Health Research Authority^3^ has recently adopted the same requirement for all interventional clinical trials conducted in the United Kingdom. The European Union Clinical Trial Regulation (no. 536/2014) makes it obligatory for sponsors of Clinical Trials of Investigational Medicinal Products (CTIMPs) to upload the summary results of those trials onto the European Union Clinical Trial Register within a maximum of 12 months after trial completion^4^ (Box 1). However, timely reporting of clinical trial results on registries does not always happen in practice.^5,6^ Goldacre et al.^5^ estimated in 2018 that only 49.5% of trials on the European Union (EU) Clinical Trial Register reported results as required; non-commercial sponsors such as universities performed especially weakly.

**Box 1. What are CTIMPs?**

The UK Health Research Authority defines a Clinical Trial of a Medicinal Investigational Product (CTIMP) as ‘an investigation in human subjects which is intended to discover or verify the clinical, pharmacological and/or other pharmacodynamic effects of one or more medicinal products, identify any adverse reactions or study the absorption, distribution, metabolism and excretion, with the object of ascertaining the safety and/or efficacy of those products’.^7^ The Health Research Authority includes pharmacokinetic studies in this definition. All CTIMPs conducted in the European Union must register on the EU Clinical Trial Register and upload their summary results within 12 months of completion.^4,8^

In the past 3 years, we have evaluated clinical trial policies of UK universities, tracking their clinical trial registration and reporting policies as well as their reporting performance on the EU Clinical Trial Register and the American ClinicalTrials.gov trial registry^9–11^. This took place within the framework of the Universities Allied for Essential Medicines UK’s Global Health Report Cards,^12^ which evaluate the policies and performance of universities in the United Kingdom with regard to global health equity. These regular assessments look primarily at university policies around open access publishing and technology transfer, but since 2018, a section on clinical trial transparency has been added. The first report was published in 2018, showing that, at the time, 1639 clinical trials sponsored by UK universities were missing results on the EU Clinical Trial Register and ClinicalTrial.gov trial registries more than 12 months after trials ended.^9^ Following pressure from civil society organisations such as TranspariMED, the AllTrials campaign, Universities Allied for Essential Medicines UK, as well as researchers at the University of Oxford, the lack of timely clinical trial reporting by university sponsors drew attention on a national level. In 2018, the UK House of Commons’ Science and Technology Committee, a standing parliamentary committee, launched an enquiry into clinical trial transparency that discussed persistent gaps in trial reporting and called for remedial action to be taken, including the imposition of sanctions for non-compliant sponsors.^13^ In January 2019, the Chair of the committee sent letters to more than fifty UK universities and other public bodies sponsoring clinical trials, requesting them to upload any missing CTIMP results onto the EU Clinical Trial Register by summer of the same year.^5,9,13^ The letter announced that after the summer, a public hearing would take place to follow up on sponsors’ progress, with persistently non-compliant sponsors invited to explain their performance.^14^ The aim of this study is to document changes in the clinical trial policies and Clinical Trials of Investigative Medicinal Product reporting performance of 20 major UK universities following that intervention. We have reported universities’ evolution of reporting performance for clinical trials registered on the ClinicalTrials.gov registry elsewhere.^11^

## Methods

The selection procedure of Universities Allied for Essential Medicines UK’s Global Health Report Cards project is extensively described by Gotham et al.^12^ We first selected the 25 UK universities receiving the largest sums of public medical research funding, using Medical Research Council funding obtained in 2017–2018 as a proxy indicator. We then added two universities that had been assessed in a 2014 Global Health Report Cards project. For the purpose of this study, we then excluded universities with very small drug trial portfolios, that is, less than 10 trials listed on EU Clinical Trial Register. We filed Freedom of Information requests in June 2018 and again in June 2020 via the online platform WhatDoTheyKnow.com to obtain universities’ clinical trial registration and reporting policies. Policies were assessed against the following WHO best practices and Declaration of Helsinki requirements:^1,2^ (1) all clinical trials are required to be registered; (2) only some clinical trials are required to be registered (e.g. only CTIMPs); (3) clinical trials are required to be registered before the first subject receives the first medical intervention; (4) registry entries are required to be regularly updated; (5) clinical trials are required to post summary results within 12 months of their primary completion date; (6) all clinical trials are required to post summary results; (7) only some clinical trials are required to post summary results (e.g. only CTIMPs). Only policies that were made publicly available through universities’ websites or in response to Freedom of Information requests were assessed. Two reviewers independently assessed each university’s policies. Disagreements were resolved by consensus following discussions involving a third team member. To evaluate universities’ reporting performance on EU Clinical Trial Register, the European registry for CTIMPs, we used the EU Trials Tracker (https://eu.trialstracker.net/), an online tracking tool developed by Goldacre et al.^5^ The tracker assesses trial sponsors’ regulatory compliance by identifying trials completed more than 12 months ago and checking whether summary results for these are available on the EU Clinical Trial Register as required.

## Results

Universities’ mean policy strength increased from 2.8 to 4.6 (out of a maximum possible 7 points) between 2018 and 2020. Universities’ mean reporting performance on the EU Clinical Trial Register increased from 29% to 91% between 2018 and 2021.

### Policies

Mean policy strength across the 20 universities changed from 2.8 to 4.6 out of a maximum of 7 points between 2018 and 2020, although not all universities changed their policies during the study period (Table 1 and Table 2). For the 13 universities that made publicly verifiable changes to their policies, mean policy strength increased from 1.8 to 4.9. In 2018, no university’s policies fully incorporated relevant WHO best practices and Declaration of Helsinki stipulations; in 2020, 6/20 universities verifiably fully met those benchmarks. Nonetheless, some gaps remained in 2020. The policies of 6/20 universities did not reflect the regulatory requirement (and WHO best practice) of uploading the results of CTIMPS onto the EU Clinical Trial Register after trial completion. The policies of 6/20 universities did not require all clinical trials to be registered; in addition to falling short of WHO best practices and Declaration of Helsinki stipulations, this also falls short of national UK ethics requirements.^3,14^

**Table 1.** Overview of policy strength and clinical trial reporting performance on EU Clinical Trial Register of 20 UK universities receiving most Medical Research Council funding in the year 2017–2018, ordered by number of due trials on EU Clinical Trial Register. Due trial is a trial with a completion date of more than 12 months ago. Policy strength is based on Freedom of Information requests filed in June 2018 and June 2020 was assessed according to criteria based on the World Health Organization (WHO) statement on clinical trial transparency. The following criteria were assessed (1) all clinical trials are required to be registered; (2) only some clinical trials are required to be registered (e.g. only CTIMPs); (3) clinical trials are required to be registered before the first subject receives the first medical intervention; (4) registry entries are required to be regularly updated; (5) clinical trials are required to post summary results within 12 months of their primary completion date; (6) all clinical trials are required to post summary results; (7) only some clinical trials are required to post summary results (e.g. only CTIMPs). Policies that did not change between 2018 and 2020 or where not made available through Freedom of Information requests in 2020 are indicated with an asterisk and were not evaluated. The clinical trial reporting performance data were downloaded from the EU Trials Tracker on 1 October 2018 and 28 June 2021, respectively. The last update of the EU Trials Tracker website before the download in June 2021 occurred on the 5th of June 2021. For further information on the methodology, see Keestra et al.^9^ and forthcoming publication of the Universities Allied for Essential Medicines UK Global Health Report Cards. Full data set available on request and at https://zenodo.org/record/5591899#.YXKWr9lBw-R.

University name	Policy strength 2018	Policy strength 2020	Reporting performance 2018	Reporting performance 2021
Imperial College London	3/7	3/7*	5/19 (26%)	93/97 (96%)
University College London	1/7	3/7	13/20 (65%)	81/85 (95%)
University of Oxford	3/7	6/7	21/26 (81%)	65/78 (83%)
King’s College London	4/7	4/7*	6/12 (50%)	69/69 (100%)
University of Dundee	4/7	7/7	50/61 (82%)	67/68 (99%)
University of Nottingham	2/7	7/7	1/17 (6%)	51/54 (94%)
University of Birmingham	6/7	7/7	2/13 (15%)	52/52 (100%)
University of Leeds	0/7	7/7	8/14 (57%)	50/51 (98%)
University of Edinburgh	5/7	5/7*	2/8 (25%)	36/41 (88%)
Queen Mary University of London	0/7	4/7	6/14 (43%)	39/39 (100%)
Cardiff University	3/7	7/7	2/10 (20%)	26/36 (72%)
University of Liverpool	2/7	2/7*	0/1 (0%)	19/25 (76%)
University of Cambridge	4/7	4/7*	0/1 (0%)	19/23 (83%)
University of Bristol	3/7	3/7*	1/3 (33%)	13/16 (81%)
University of Glasgow	4/7	4/7	1/5 (20%)	14/14 (100%)
The University of Manchester	3/7	3/7	0/2 (0%)	10/13 (77%)
University of Leicester	6/7	6/7*	0/2 (0%)	12/12 (100%)
University of Southampton	0/7	0/7	0/0 (NA)	7/8 (88%)
London School of Hygiene and Tropical Medicine	1/7	2/7	0/1 (0%)	7/8 (88%)
University of Warwick	Not publicly available	7/7	1/3 (33%)	7/7 (100%)
Average	2.8/7	4.6/7	29%	91%

**Table 2.** Clinical trial policy ratings of 20 UK universities receiving most MRC funding in the year 2017–2018, ordered by number of due trials on EU Clinical Trial Register. Policy strength is based on Freedom of Information Requests filed in June 2018 and June 2020 was assessed according to criteria based on the World Health Organization (WHO) Joint statement on public disclosure of results from clinical trials? Where policies were shared with local NHS trusts, policies were rated as if university policies, but where universities did not have a specific policy and only referred to the Health Research Authority guidelines, the university was awarded 0 points in the scoring of the policy. Policies were assessed by two independent reviewers and disagreements resolved by a third reviewer. Blue indicates a score of one point on the item, orange indicates a zero score on the item. Black indicates no policy change occurred between 2018 and 2020 (based on universities’ responses via Freedom of Information requests) or that the university policy has not been made publicly available and therefore cannot be assessed. Blacked out policies were therefore not assessed and did not contribute to the ratings.

University name	Year	The university has a policy that requires all clinical trials to be registered.	The university has a policy that requires some clinical trials (e.g. CTIMPs) to be registered.	The university has a policy that requires clinical trials to be registered before the first subject receives the first medical intervention	The university has a policy that requires registry entries to be regularly updated	The university has a policy that requires clinical trials to post summary results within 12 months of their primary completion date.	The university has a policy that requires all clinical trials to post summary results.	The university has a policy that requires some clinical trials (e.g. CTIMPs) to post summary results.
Cardiff University	2018	0	1	1	1	0	0	0
	2020	1	1	1	1	1	1	1
Imperial College London	2018	1	1	1	0	0	0	0
	2020							
King's College London	2018	0	1	1	0	1	0	1
	2020							
London School of Hygiene and Tropical Medicine	2018	0	1	0	0	0	0	0
	2020	0	1	0	1	0	0	0
Queen Mary University of London	2018	0	0	0	0	0	0	0
	2020	1	1	0	0	0	1	1
The University of Manchester	2018	1	1	1	0	0	0	0
	2020	1	1	1	0	0	0	0
University College London	2018	0	1	0	0	0	0	0
	2020	0	1	0	0	1	0	1
University of Birmingham	2018	1	1	1	0	1	1	1
	2020	1	1	1	1	1	1	1
University of Bristol	2018	1	1	1	0	0	0	0
	2020							
University of Cambridge	2018	0	1	1	0	1	0	1
	2020							
University of Dundee	2018	0	1	1	0	1	0	1
	2020	1	1	1	1	1	1	1
University of Edinburgh	2018	1	1	1	0	1	0	1
	2020							
University of Glasgow	2018	1	1	1	1	0	0	0
	2020	1	1	1	1	0	0	0
University of Leeds	2018	0	0	0	0	0	0	0
	2020	1	1	1	1	1	1	1
University of Leicester	2018	1	1	1	0	1	1	1
	2020							
University of Liverpool	2018	0	0	0	0	1	0	1
	2020							
University of Nottingham	2018	0	1	1	0	0	0	0
	2020	1	1	1	1	1	1	1
University of Oxford	2018	0	1	0	0	1	0	1
	2020	1	1	1	1	1	0	1
University of Southampton	2018	0	0	0	0	0	0	0
	2020	0	0	0	0	0	0	0
University of Warwick	2018							
	2020	1	1	1	1	1	1	1

### Reporting performance

Between October 2018 and June 2021, the universities’ CTIMP reporting performance on the EU Clinical Trial Register increased from a mean of 29% to 91% (Table 1). By June 2021, all 20 universities had reported more than 70% of their due CTIMP results on the registry, and 5/20 universities had a perfect reporting rate of 100%. Nonetheless, in June 2021, a total of 74 CTIMPs sponsored by the universities in our cohort were still missing results on the registry, despite having completed more than 12 months ago, in violation of regulatory requirements.

## Interpretation

In this assessment of clinical trial transparency at the top publicly funded universities in the United Kingdom between 2018 and 2021, we found significant improvements in performance but only minor improvement in policies. Whereas in October 2018 university sponsors on average only had reported the results of 29% of the trials subject to regulatory disclosure requirements, the mean reporting performance had dramatically increased to 91% by June 2021. At the time of our assessment, UK universities all have a reporting performance of more than 70%, although 74 studies are still missing results. Major UK universities’ CTIMP reporting rate of 91% on average compares extremely favourably with the average estimated CTIMP reporting rate of 28% found in a recent assessment of 26 major European research institutions.^15^ Presumably, this stronger performance in the United Kingdom is largely due to UK parliament’s focus on the issue and the letters sent to UK universities in early 2019, highlighting the potential of political engagement to drive improvements in clinical trial transparency. It should be noted though that there was also significant engagement from civil society and academia with the issue of clinical trial transparency during the same period. The hypothesis that political engagement drove the increase in CTIMP reporting performance on the EU Clinical Trial Register is supported by data showing that UK universities during the same time period did not extend their reporting efforts to other types of clinical trials listed on different trial registries; we found in October 2020 that only 10.2% of trials sponsored by UK universities had reported tabular summary results on the US-based ClinicalTrials.gov registry.^11^ The committee’s letter only referred to CTIMPs, so universities largely focused their efforts on these trials only; also there is no regulatory requirement to upload the results of non-CTIMPs onto trial registries.

That improvements in reporting performance appear confined to one type of trial (CTIMPs) and one registry (EU Clinical Trial Register) is a matter of concern, as are the persistent gaps in most universities’ policies. Despite improvements in reporting results on EU Clinical Trial Register in practice, policy changes at an institutional level are lagging in comparison even among universities that perform well in reporting due trials. The policies of 6/20 universities now verifiably fully meet relevant regulatory requirements and global best practice benchmarks, but the 14 other universities assessed in this study still need to follow suit. The contrast between the dramatic improvement in CTIMP reporting rates and the more modest improvement in policies may be due to UK universities in 2019 making significant efforts to retrospectively report missing CTIMP results within the brief time frame set by UK parliament. Universities’ efforts may not always have extended to addressing the underlying problem of gaps in their related policies.

This study has limitations. First, we only captured changes in policy documents, but not other potentially relevant changes. For example, Imperial College London did not change any of their policies, but state in response to the Freedom of Information request that they are now offering regular workshops to principal investigators on clinical trial registration and reporting on the EU Clinical Trial Register, and send reminders to researchers when trial results are due. Such changes are not captured by this study, but may have had an important influence on improving universities' reporting performance since 2018. Second, universities could have registered trials on several trial registries but only uploaded summary results onto one registry. There is currently no tracking tool to verify whether summary results missing on one trial registry are available on another registry, on a national database, or in the academic literature. Thus, this study cannot distinguish between CTIMPs missing results on EU Clinical Trial Register, and CTIMPs that remain completely unreported. Third, our methodology for assessing reporting performance cannot identify unreported trials that have not been correctly marked as ‘completed’ on the EU Clinical Trial Register.^6^ Fourth, we assessed the policies of universities only. This is a significant limitation as many UK university-led clinical trials are formally sponsored by university hospital trusts rather than by the universities themselves.

Going forward, the UK Health Research Authority’s #MakeItPublic strategy, which seeks to ensure that every clinical trial conducted in the United Kingdom is prospectively registered and makes its results public, will most likely improve clinical trial reporting across all trial registries.^3^ This strategy was developed in response to the 2018 parliamentary enquiry’s recommendations. Following Brexit, no new CTIMPs by UK sponsors can be registered on the EU Clinical Trial Register. The UK’s national medicines regulator, the Medicines and Healthcare products Regulatory Agency, now recommends the use of other international registries such as ClinicalTrials.gov to register CTIMPs instead.^16,17^ Most recently, the National Health Service (NHS) Health Research Authority^18^ announced a partnership with the ISRCTN registry, which will automatically preregister all new clinical trials on this international clinical trial database, which is recognised by the WHO as fulfilling the requirement of registration before the first participant receives an intervention. However, UK sponsors continue to have the obligation to upload results for older CTIMPs onto whichever registry they were registered on once completed.

UK non-commercial sponsors, including universities, currently have the highest reporting performance rates on the EU Clinical Trial Register compared to other European non-commercial sponsors of CTIMPs.^19^ Based on the UK’s positive experience, we suggest enlisting parliamentarians and other political decision-makers in efforts to improve clinical trial registration and reporting, including through sanctions for non-compliant researchers and/or institutions.^19,20^ To ensure that patients receive the most optimal treatment based on the latest clinical evidence, interventions such as these are vital for improving clinical trial transparency globally.
